# Comparison of the effect of breast pump stimulation and oxytocin administration on the length of the third stage of labor, postpartum hemorrhage, and anemia: a randomized controlled trial

**DOI:** 10.1186/s12884-018-1832-z

**Published:** 2018-07-07

**Authors:** Elham Dashtinejad, Parvin Abedi, Poorandokht Afshari

**Affiliations:** 1 Midwifery Department, Reproductive Health Promotion Research Center, Ahvaz Jundishapur University of Medical Sciences, Ahvaz, Iran; 2 Community Nutrition, Midwifery Department, Menopause Andropause Research Center, Ahvaz Jundishapur University of Medical Sciences, 13th East Kianpars Ave, 1st Eastern Maroon, No:46, Ahvaz, Iran

**Keywords:** Breast stimulation, Postpartum hemorrhage, Hemoglobin, Hematocrit

## Abstract

**Background:**

This study aimed to compare the effect of breast pump stimulation with that of oxytocin administration regarding the duration of the third stage of labor, postpartum hemorrhage, and anemia after delivery.

**Methods:**

In this study, 108 women were randomly assigned to two groups of breast pump stimulation (*n* = 54) and oxytocin administration (*n* = 54). Women in the breast stimulation group received breast pump stimulation (10 min intermittently for each breast with a negative pressure of 250 mmHg), while the women in the oxytocin (control) group received an infusion of 30 IU oxytocin in 1000 mL of Ringer’s serum with a maximum rate of 10 mL infusion per min after delivery. The duration of the third stage of labor, blood loss during the third stage of labor and 24 h after delivery, hemoglobin and hematocrit (before and 24 h after delivery), after-birth pain, and the number of breastfeedings during the 24 h after delivery were recorded. The data were analyzed using the chi-square test, independent t-test, and Wilcoxon test.

**Results:**

The mean duration of the third stage was 5 ± 1.97 and 5.4 ± 2.5 min in the breast stimulation and women that received intravenous oxytocin respectively (*p* = 0.75). Most participants had mild postpartum hemorrhage (98.1 and 96.2% in the breast stimulation and women that received intravenous oxytocin, respectively, *p* = 0.99). Although hemoglobin and hematocrit levels significantly decreased in both groups 24 h after delivery, there was no significant difference between both groups regarding both parameters. After-birth pain was significantly lower and the number of breastfeeding during the 24 h after delivery was significantly more in the breast stimulation group compared to the control group.

**Conclusions:**

Our results demonstrated no differences between breast pump stimulation and oxytocin administration regarding the duration of the third stage of labor, postpartum hemorrhage, anaemia, after-birth pain, and the number of breastfeedings during the 24 h after delivery.

**Trial registration number:**

The study protocol was registered in the Iranian Randomized Controlled Trial Registry (Ref. No.: IRCT2015050722146N1; Registration date: 2015–11-04). The study was registered prospectively and the enrollment date was 23/8/2015.

**Electronic supplementary material:**

The online version of this article (10.1186/s12884-018-1832-z) contains supplementary material, which is available to authorized users.

## Background

Postpartum hemorrhage (PPH) is one of the most life-threatening events during labor and birth, and its prevalence ranges from 6 to 10.5% [1]. Uterine atony is the most common cause of PPH, which mostly occurs following the expulsion of the placenta in the third stage of labor [2]. PPH increases the risk of hypotension, anemia, fatigue, blood transfusion and hemorrhagic shock among women [3].

There are two methods for managing the third stage of labor: physiologic and active management. In active management, 5 or 10 IU oxytocin is administered intramuscularly (IM) after the expulsion of the neonate’s shoulder or after the delivery of the placenta [4]. The World Health Organization recommends the use of uterotonic agents, preferably oxytocin, for all births during the third stage of labor; however, there is moderate-quality evidence supporting this recommendation [5]. Prendiville et al., in a systematic review including five studies, showed that while active management of the third stage of labor, compared to expectant management, could reduce PPH exceeding 500 mL, maternal blood loss, and prolonged third stage of labor, it could cause maternal nausea, vomiting, and increased blood pressure [6]. Begley et al., in a systematic review of seven studies, found that active management of the third stage of labor could significantly decrease the risk of PPH exceeding 1000 mL in women with a mixed risk of excessive bleeding but could increase the risk of adverse effects [7]. Some maternal complications associated with the administration of oxytocin include nausea, vomiting, increased blood pressure, and adverse cardiovascular response [8]. Water intoxication is another side effect of oxytocin, especially when it administered continuously by infusion [9].

The effect of suckling on the length of the third stage of labor and PPH was assessed in a study by Niroomanesh et al. Their results showed that the suckling group had a significantly longer third stage of labor compared to the oxytocin group. The number of sanitary pads used in the suckling group was significantly more than that used in the oxytocin group (11.72 vs. 10.58, *p* < 0.001) [10]. Contrastingly, Narenji et al. found that the mean blood loss in the third stage of labor was 167.14 ± 306.7, 219.7 ± 329.79, and 220.2 ± 331.2 mL in the expectant management, breastfeeding, and IM injection of oxytocin groups, respectively (*p* > 0.05). However, the length of the third stage of labor was lower in the breastfeeding and oxytocin groups compared to the expectant management group (*p* > 0.05) [11]. Suckling, nipple or breast stimulation or breastfeeding can increase the release of endogenous oxytocin [12]. Moreover, studies have shown that nipple stimulation or breastfeeding cause the secretion of oxytocin in a pulsatile manner involving 3 to 4 s secretions of oxytocin into the blood-stream every 5 to 15 min [13].

A systematic review including four studies (4608 women) was conducted comparing breast-feeding and nipple stimulation for the reduction of PPH or bleeding in the third stage of labor. The results revealed a lack of sufficient data for evaluating the impact of nipple stimulation for the reduction of PPH and indicated that more studies with a sufficient sample size and of high quality were needed to ascertain this impact [14]. Most trials have demonstrated the positive effect of the active management of labor in women with moderate or high risks of PPH, but not in those at low risk of bleeding [7]. Thus, the present study aimed to compare the effect of breast pump stimulation with that of oxytocin administration with respect to the length of the third stage of labor, PPH and anemia in women at low risk for PPH.

## Methods

This was a randomized controlled trial that included 108 women who were randomly assigned to a breast pump stimulation (*n* = 54) and oxytocin administration (*n* = 54) group at the beginning of the third stage of labor.

This study was approved by the Ethics Committee of Ahvaz Jundishapur University of Medical Sciences (Ref No: IR.AJUMS.REC.1394.86), and the study protocol was registered in the Iranian Randomized Controlled Trial Registry (Ref. No.: IRCT2015050722146N1). All women provided written informed consent before data collection. The inclusion criteria were as follows: age 18 to 35 years old, singleton fetus, gestational age 37 to 42 weeks, normotensive women, first and second pregnancy, hemoglobin before delivery > 11 g/dL, body mass index at first trimester of pregnancy 20 to 30 kg/m^2^, estimated weight of fetus 2500 to 4000 g and normal vaginal delivery. Women with placenta abruption, placenta previa, obstructed labor, medical problems, history of cesarean section, need for induction or augmentation of labor, history of third-stage bleeding, and history of abortion were excluded from the study.

The following formula was used for sample size calculation [15]:

$$ n=\frac{\left({S}_1^2+{S}_2^2\right)}{\left(\overline{X_1}+\overline{X_2}\right)}f\left(a,\beta \right)=54 $$

$$ \alpha =0.05 $$

$$ \beta =0.2\kern0.6em \overline{X_1}=5.4\kern0.6em \overline{X_2}=8.1\kern0.6em {\mathrm{S}}_1=4.1\kern3.959998em {\mathrm{S}}_2=5.7 $$

### Randomization

A random table generated by the Excel software (Microsoft, Redmond, WA, USA) was used for randomization. Eligible women were recruited in the intervention or control groups at a ratio of 1:1. Considering allocation concealment, the receptionist in the labor ward was responsible for providing each participant with a number from the randomization table and she, thereafter, referred the patient to the researcher for collecting data.

### Setting

Sina Hospital was selected for the conducting of the study. This governmental hospital is located in Ahvaz (the capital city of the Khuzestan province located in Southwest Iran). The average number of vaginal deliveries in Sina hospital is 350 per month. Because of the high number of deliveries and limitation in the number of labor delivery and recovery rooms (LDRs) skin-to-skin touch immediately after birth is not performed in this hospital.

### Measures

A demographic questionnaire and check list were used to gather data. The body mass index at the first trimester of pregnancy was recorded. Gestational age was calculated based on the first-trimester ultrasonography and last menstrual period. Blood pressure was measured while the woman was in a sitting position. Hemoglobin and hematocrit were measured in 2-mL venous blood samples that were drawn at the time of admission to hospital and 24 h after delivery and sent to Sina Hospital laboratory. Data regarding neonatal status, episiotomy and perineal tears were recorded on the checklist. The neonate head circumference, weight, sex, APGAR score at the first and fifth minute after delivery, and the need for resuscitation were also recorded. PPH was measured using the Higham chart [16]. All participants in the two groups received special sanitary pads during the postpartum period. The amount of the blood in the sanitary pads was measured in centimeters. A small amount of blood in the sanitary pads was multiplied by a factor of 1, moderate amount of blood by a factor of 5, and large amount of blood by a factor of 20. The expulsion of clots was also recorded. All measurements regarding PPH continued 24 h after delivery for both groups.

The intensity of after-birth pain, mother’s satisfaction, success in the first breastfeeding, and number of neonate feedings 24 h after delivery were also recorded. The intensity of pain 24 h after delivery was measured using the visual analog scale (VAS) (every 2 h). Any maternal complications such as abnormal bleeding and the need for oxytocin or ergometrine were also recorded.

### Intervention

The two groups did not receive any intervention until the completion of the second stage of labor. For women in the breast-stimulation group, immediately after the delivery of the baby, a shield was placed on each breast and the breasts were stimulated intermittently for 10 min (20 min for both breasts). This shield was connected to the pump with negative pressure up to 250 mmHg. The pump used in this study (Medela, Zug, Switzerland), had a three-stage suction rhythm like a baby sucking.

Women in the control group received 30 IU of oxytocin in 1000 mL of Ringer’s serum with a maximum rate of 10 mL infusion per min. Women in the intervention group received Ringer’s serum without oxytocin. Two skilled midwives performed all deliveries. One of the investigators (ED) performed all measurements after delivery. The third stage of labor was measured using a chronometer. Third-stage labor bleeding was estimated visually. Hemoglobin and hematocrit levels were determined before delivery and 24 h after delivery using 2-mL venous blood samples of participants.

### Statistics

All data were analyzed with SPSS version 22 (IBM, Armonk, NY, USA) and the normality of data was determined using the Kolmogorov-Smirnov test. Categorical data were analyzed using the chi-square test, while continuous data in the two groups were compared using an independent t-test and Mann-Whitney test for normally and non-normally distributed data respectively. The Wilcoxon test was used for comparing data before and after the intervention. *p* < 0.05 was considered significant.

## Results

**Fig. 1:**
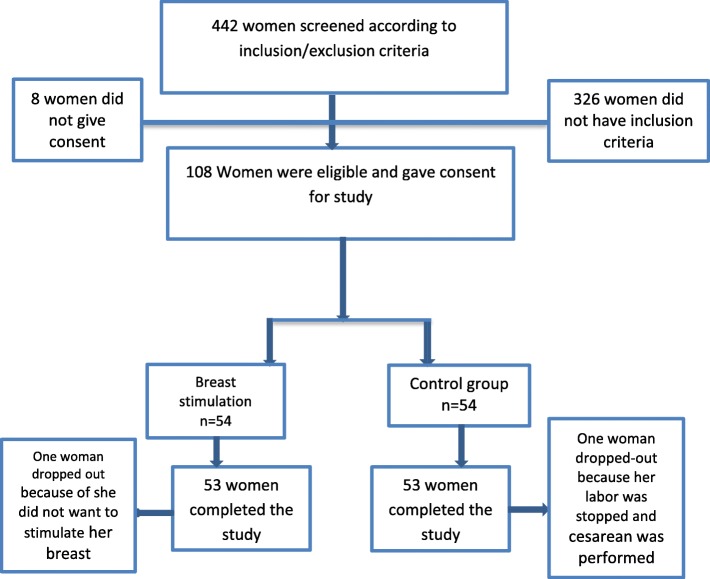
Flow diagram of recruitment and retention of participants in the study

Fifty-three women each in the breast-stimulation and control groups completed the study. The reasons for withdrawal are outlined in Fig. 1. All data are presented in Additional file 1.

The mean participant age was 22.9 ± 3.73 and 23 ± 4.19 years in the breast-stimulation and control groups, respectively. The body mass index of most participants was with in the normal range. Most participants were primigravida (71.6 vs. 62.2% in the breast stimulation vs. control groups). Other socio-demographic features of the participants are listed in Table 1.

**Table 1:** Socio-demographic characteristics of women in two groups of breast stimulation and control

Variables	Breast stimulation *n* = 53	Control group *n* = 53	*P* value
Mean ± SD or N (%)			
Age (y)	22.9 ± 3.73	23 ± 4.19	0.84
Gestational age(wks)	39.1 ± 1.22	39.01 ± 1.14	0.71
Body mass index (kg/m^2^)	24.8 ± 2.6	25.05 ± 3.04	0.51
Education			
Primary school	17(32.07)	8(15.09)	0.071
Secondary school	17(32.07)	15(28.3)	
Diploma	17(32.07)	24(45.2)	
University education	2(3.77)	6(11.3)	
Number of pregnancy			
First	38(71.6)	33(62.2)	0.31
Second	15(28.3)	20(37.7)	
Economic situation			
Weak	13(24.5)	8(15.09)	0.47
Moderate	26(49)	31(56.6)	
Good	14(26.4)	15(28.3)	

The systolic and diastolic blood pressure levels of all participants were within the normal range. The systolic blood pressure was 113.5 ± 7 mmHg and decreased to 104.6 ± 7 mmHg after delivery in the breast stimulation group (*p* < 0.001). The systolic blood pressure also decreased from 113 ± 7 mmHg to 103 ± 6.39 mmHg in the control group (*p* < 0.001).

The mean of hemoglobin significantly decreased from 12.46 ± 1 g/dL to 10.96 ± 0.95 g/dL in the breast stimulation group and from 12.5 ± 1.27 g/dL to 10.87 ± 1.17 g/dL in the control group (*p* < 0.001). The mean of hematocrit significantly decreased in the breast stimulation (37.12 ± 3.11 to 33.02 ± 2.63, *p* < 0.001) and control (37.5 ± 3.89 to 32.87 ± 3.52, *p* < 0.001) groups 24 h after delivery, compared to baseline. However, there was no significant difference between the two groups regarding systolic, diastolic blood pressure, hematocrit and hemoglobin level (Table 2).

**Table 2:** Blood indices and blood pressure of participants in the breast stimulation and control groups before and after intervention

Variables	Breast stimulation *n* = 53			Control *n* = 53		
	Mean ± SD					
	Before	After	Mean difference	Before	After	Mean difference
Systolic blood pressure (mmHg)	113.5 ± 7	104.6 ± 7*	− 8.99	113 ± 7	103 ± 6.39*	− 10
Diastolic blood pressure (mmHg)	74.1 ± 4.9	63.7 ± 5.5*	− 10.46	73.8 ± 5.6	63.5 ± 5.1*	− 10.37
Hemoglobin (gr/dL)	12.46 ± 1	10.96 ± 0.95*	− 1.50	12.5 ± 1.27	10.87 ± 1.17*	− 1.65
Hematocrit (%)	37.12 ± 3.11	33.02 ± 2.63*	− 4.10	37.5 ± 3.89	32.87 ± 3.52*	− 4.67

*P value within groups is significant < 0.001

All *p* values before and after intervention between groups were not significant

All *p* values regarding mean differences were not significant

The third stage duration did not significantly differ between the two groups (5 ± 1.97 vs. 5.4 ± 2.5 min in the breast stimulation and control groups respectively, *p* = 0.75). The placenta was mainly expulsed spontaneously in both groups (61.1 and 51.9% in the breast stimulation and control groups, respectively; *p* = 0.54). Most participants did not have perineal tears (92.4 and 81.13% in the breast stimulation and control groups, respectively; *p* = 0.08).

Most participants did not have PPH, and their bleeding was in the normal range (< 500 mL) (96.2% in the breast stimulation group and 92.4% in the control group, *p* = 0.67). The number of women with moderate bleeding (500–1000 mL) in the control group was almost twice that of women in the breast stimulation group. Most mothers in the breast stimulation group were satisfied with the method (83.01%), while only 5.66% of women in the control group were satisfied with oxytocin administration. The number of breastfeedings during the 24 h after delivery in the breast stimulation group was more than that in the control group (9.14 ± 1.41 vs. 8.51 ± 2.16), but this difference was not significant (*p* = 0.16). Most mothers in the breast stimulation group had mild after-birth pain (90.5%), unlike in the control group (26.4%) (*p* < 0.001) (Table 3).

**Table 3:** Main delivery outcomes in the breast stimulation and control groups

Variables	Breast stimulation *n* = 53	Control group *n* = 53	*P* value
	Mean ± SD or N (%)		
Third stage length (min)	5 ± 1.97	5.4 ± 2.5	0.75
Number of breast feeding in 24 h	9.14 ± 1.41	8.51 ± 2.16	0.16
Placenta expulsion			
Spontaneously	33(61.1)	28(51.9)	0.54
With fundus compression	16(29.6)	18(33.3)	
With controlled cord traction	5(9.3)	8(14.8)	
Neonatal complication			
Yes	0	1(1.88)	0.49
No	53(100)	52(98.11)	
Perineal tears			
Yes	4(7.5)	10(18.8)	0.08
No	49(92.4)	43(81.13)	
Episiotomy			
Yes	47(88.67)	43(81.1)	0.27
No	6(11.32)	10(18.8)	
First breastfeeding			
Successful	51(96.2)	46(86.7)	0.16
Unsuccessful	2(3.77)	7(13.2)	
Bleeding in the third stage of labour (mL)			
< 500	51(96.2)	49(92.4)	0.67
500–1000	2(3.77)	4(7.5)	
Postpartum hemorrhage according to Higham chart			
Mild	52(98.1)	51(96.2)	0.99
Moderate	1(1.8)	2(3.77)	
Mother satisfaction			
Low	1(1.88)	8(15.09)	< 0.001
Moderate	8(15.09)	42(79.2)	
High	44(83.01)	3(5.66)	
Postpartum pain			
Mild	48(90.5)	14(26.4)	< 0.001
Moderate	5(9.4)	30(56.6)	
Severe	0	9(16.9)	

In this study, two women in the breast stimulation group had bleeding exceeding 500 ml. These women received 10 IU oxytocin (IM) and 40 IU oxytocin by infusion. In the control group, four women had bleeding exceeding 500 ml. Two received 10 IU oxytocin (IM) and 40 IU oxytocin by infusion. One received 80 IU oxytocin (infusion), 10 mg methergine (IM), and 800 μg misoprostol (sublingual), and the last received 10 IU oxytocin (IM) and 80 IU by infusion. The last two women had nausea and vomiting. In this study, four women in the breast stimulation group and 18 women in the control group received analgesics for after-birth pain. There were no significant differences between both groups in neonatal characteristics, including APGAR score, newborn weight, head circumference, and sex of the newborn (Table 4).

**Table 4:** Neonatal characteristics in the breastfeeding and control groups

Variables	Breastfeeding *n* = 53	Control *n* = 53	*P* value
	Mean ± SD		
First minute APGAR	9	8.9 ± 0.42	0.15
Fifth minute APGAR	10	9.98 ± 0.13	0.31
Newborn weight (gr)	3226 ± 375	3146 ± 353	0.33
Head circumference of newborn (cm)	33.9 ± 1.1	33.9 ± 0.9	0.95
Sex of new born			
Female	27(50.9)	26(49.05)	0.70
Male	26(49.05)	27(50.9)	

## Discussion

This study aimed to compare the effect of breast pump stimulation with that of oxytocin administration regarding the length of the third stage of labor, PPH, and anemia. The results of the present study showed no significant difference between the groups regarding the length of the third stage of labor. Additionally, this study revealed that the number of women with moderate bleeding (500–1000 mL) in the control group was almost twice that in the breast stimulation group; however, the two groups did not show any significant difference regarding bleeding in the third stage of labor. Moreover, the two groups did not show any significant difference regarding hemoglobin and hematocrit levels and mean bleeding (measured by the Higham scale) 24 h after delivery.

Bullough et al. conducted a study on three groups of women: the women in one group were shown how to squeeze their nipples for 15 min (*n* = 6), another group received syntometrine injection (*n* = 3), and the other group (*n* = 5) received physiologic management of the third stage of labor. The results showed that the syntometrine group had the least bleeding (mean: 83 mL) compared to the nipple stimulation (mean: 166 mL) and physiologic management (mean: 257 mL) group. The authors concluded that breastfeeding in low-resource areas can be an effective means for decreasing PPH [17]. These results for mean bleeding are not in line with our results, as we found that the breast stimulation and oxytocin groups did not show any significant difference regarding blood loss in the postpartum period. The reason for this discrepancy may be the considerably smaller number of participants in the study by Bullough et al.

A study by Narenji et al., involving three groups of women (*n* = 150), assessed the effect of breastfeeding on the length of the third stage of labor and PPH. One group received 10 IU oxytocin (IM) immediately after delivery (*n* = 50), one group started breastfeeding immediately after delivery (*n* = 50), and one group did not receive any intervention (*n* = 50). The results showed that although the mean blood loss was less in the physiologic group compared to the other two groups, there was no significant difference between the three groups in the mean duration of the third stage of labor and PPH [11]. These results for the length of the third stage of labor and PPH are in line with our results.

A study by Niroomanesh et al. included 100 women who received active management of the third stage of labor and 120 women who were assigned to breast stimulation or breastfeeding after delivery. Results showed that the duration of the third stage of labor (4.42 vs. 6.08 min) and the number of sanitary pads (10.5 vs. 11.72) were significantly less in the oxytocin group [10]. These results are not similar to ours. The reason for dissimilarity may be because Niroomanesh et al. did not provide any information regarding the time of nipple sucking by the newborns. In Iran, most deliveries are performed in the lithotomy position. This position does not allow the mother to have an effective bonding and contact with the baby for successful sucking.

Our results indicated that most mothers in the breast stimulation group were satisfied with the intervention, while only a small number of women in the control group were satisfied with the procedure received. Moreover, the number of breastfeedings 24 h after delivery in the breast stimulation group was more than that in the control group. Mothers in the breast stimulation group had significantly less after- birth pain 24 h after delivery. Studies have shown that nipple stimulation or breastfeeding causes the secretion of oxytocin in a pulsatile manner, including 3 to 4 s secretion of oxytocin into the blood stream every 5 to 15 min [13]. Receiving exogenous oxytocin may interfere with the secretion of endogenous oxytocin and may delay the onset of breastfeeding and exclusive breastfeeding [18]. Studies have shown that endogenous oxytocin may decrease stress, support emotional and mental well-being and improve bonding between mother and newborn [19, 20]. Two studies have shown that women who undergo epidural anesthesia and receive oxytocin for augmentation and women who receive oxytocin for augmentation in compare to women who do not, are three times less likely to breastfeed their baby during the first four hours after delivery [21, 22].

### Strength and limitations of study

The strength of this study include the recruitment of women based on the inclusion criteria and the intensive management until 24 h after delivery. Moreover, we used a pump to stimulate the breasts to achieve the stimulation objectively. Regarding the limitations of this study, we did not have a breastfeeding group. Perhaps by recruiting women who breastfed their baby, we would have had a better comparison between breast pump stimulation and breastfeeding. In the hospital in which we conducted the study, all deliveries performed in the lithotomy position. Therefore, breastfeeding in this position is not pleasant for the mother. We did not measure the level of endogenous oxytocin in the two groups of women. This may have made the results more objective. Furthermore, we only measured hemoglobin and hematocrit 24 h after delivery. In some studies, hemoglobin and hematocrit were measured 3 to 5 days after delivery. In the third stage of labor we used visual estimation for blood loss; using other objective methods such as collecting blood in a bedpan or plastic bags may have resulted in a better estimation of blood loss, although we used the Higham chart for estimating blood loss in the postpartum period.

## Conclusion

Our results demonstrated no difference between breast stimulation and administration of oxytocin regarding the length of the third stage of labor, PPH, and anemia in low- risk women. Using breast stimulation is cost- effective and may be considered for women at low risk for PPH.

## Additional file

Additional file 1: Data of study. All data that has been collected and analyzed present in this file. (XLSX 27 kb)

## Abbreviations

IM: Intramuscular
PPH: Postpartum Hemorrhage
